# Chromosome 22q11.2 microdeletion in monozygotic twins with discordant phenotype and deletion size

**DOI:** 10.1186/1755-8166-5-13

**Published:** 2012-03-13

**Authors:** Ashutosh Halder, Manish Jain, Isha Chaudhary, Binuja Varma

**Affiliations:** 1 Department of Reproductive Biology, All India Institute of Medical Sciences, New Delhi, India; 2 The Centre for Genomic Application, 254 Okhla Industrial Area Phase III, New Delhi, India

## Abstract

We report on a pair of male monozygotic twins with 22q11.2 microdeletion, discordant phenotype and discordant deletion size. The second twin had findings suggestive of DiGeorge syndrome, while the first twin had milder anomalies without any cardiac malformation. The second twin had presented with intractable convulsion, cyanosis and cardiovascular failure in the fourth week of life and expired on the sixth week of life, whereas the first twin had some characteristic facial appearance with developmental delay but no other signs of the 22q11.2 microdeletion syndrome including cardiovascular malformation. The fluorescence in situ hybridization (FISH) analysis had shown a microdeletion on the chromosome 22q11.2 in both twins. The interphase FISH did not find any evidence for the mosaicism. The genomic DNA microarray analysis, using HumanCytoSNP-12 BeadChip (Illumina), was identical between the twins except different size of deletion of 22q11.2. The zygosity using HumanCytoSNP-12 BeadChip (Illumina) microarray analysis suggested monozygosity. This observation indicates that altered size of the deletion may be the underlying etiology for the discordance in phenotype in monozygotic twins. We think early post zygotic events (mitotic non-allelic homologous recombination) could have been played a role in the alteration of 22q11.2 deletion size and, thus phenotypic variability in the monozygotic twins.

## Introduction

The 22q11.2 microdeletion syndrome is the most common microdeletion syndrome with an estimated incidence of one in 4000 to 6,000 live births and mostly spontaneous [1-3]. The 22q11.2 microdeletion is found in patients with DiGeorge syndrome, Velocardiofacial syndrome and Conotruncal anomaly face syndrome [4]. It is characterized by wide spectrum of clinical manifestations, including craniofacial (cleft palate, velopharyngeal insufficiency), thymic and parathyroid defects as well as cardiovascular (outflow tract and aortic arch) malformations [5]. Almost all the cases result from a common deletion of the chromosome 22q11.2 locus. The FISH is the prime method for diagnosis of this microdeletion syndrome.

Several reports have mentioned phenotypic discordance between the monozygotic twins with 22q11.2 microdeletion [6-12]. No definite mechanism has been demonstrated until now for the discordant phenotype. However, somatic mosaicism, post zygotic second hit or environmental effects have been proposed. We report on a pair of monozygotic male twins with 22q11.2 microdeletion and discordant phenotype resulting from altered deletion size.

## Case Reports

### Second Twin

A four weeks old baby boy was presented in the pediatric emergency with recurrent intractable seizures, central cyanosis and unconsciousness. During evaluation in the emergency, baby had an attack of cardiopulmonary arrest. The baby was revived and kept on the ventilator. On physical examination tachycardia & systolic murmur was noted. ECG was suggestive of ventricular tachycardia. X-ray examination of chest showed cardiomegaly. Repeated blood calcium level showed hypocalcemia, despite calcium and vitamin D. The baby was suspected to have DiGeorge Syndrome with major cardiac malformation and cardiac failure. Blood sample was sent for 22q11.2 microdeletion study. The baby was expired on sixth week of life despite intensive care, including artificial life support assistance. There was no history of antenatal complications. The baby was fine for first three weeks of life. The baby was developed abnormal movements of all limbs (tonic-clonic type) along with up slanting of eyes in fourth week of life. The baby had intermittent seizures initially but became intractable in few days before referral to our hospital.

**Figure 1:**
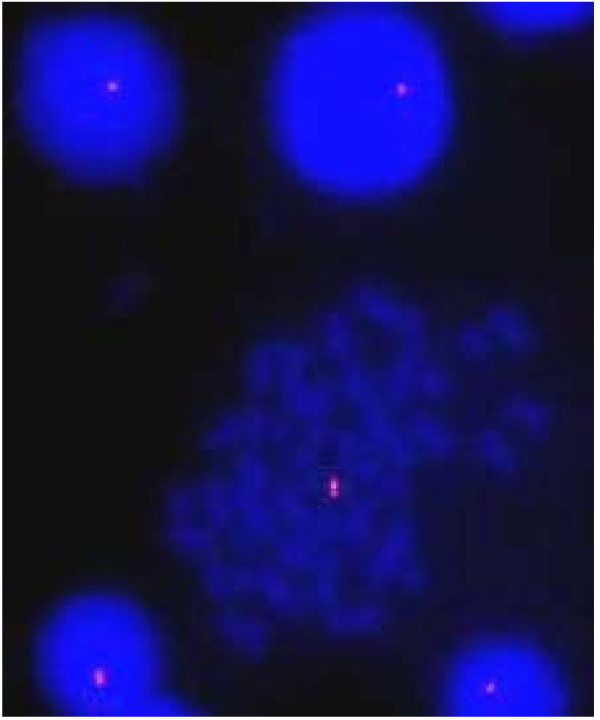
**is showing 22q11.2 FISH with deletion on interphase and metaphase cells obtained from peripheral blood lymphocyte culture**.

Since the baby had findings strongly suggestive of DiGeorge syndrome, the FISH study to detect a possible deletion in the critical 22q11.2 region was done using DNA probe specific for the 22q11.2 locus. The FISH was done using 1 ml of blood (0.5 ml heparinized for the metaphase and 0.5 ml EDTA for the interphase cells) obtained from the patient as described before [13-15]. In brief, the metaphase spreads were prepared from the phytohaemagglutinin stimulated human peripheral blood lymphocytes using standard cytogenetic technique. The interphase spreads were prepared from the blood nucleated cells (both twins), buccal cells (first twins only) and urinary cells (first twins only) after washing in phosphate buffer saline solution three times before 30 minutes of hypotonic treatment (50 mMol KCL) and the fixation in methanol:acetic acid solution (3:1 ratio). The cells (interphase & metaphase) were re-suspended in 100 ul of fresh fixative. Approximately 20 ul cell suspensions were used to prepare a slide (interphase or metaphase). FISH probe was made from the PAC clone (RP5-882J5 obtained from Uniba Biologia, Italy, by curtsy of Prof. M Rocchi). The PAC clone was grown in LB broth, DNA extracted and about one ug DNA labeled with red flurochrome (Cy3) by nick translation method. About 300 ng labeled probe per slide was used for FISH. The FISH analysis was carried out using Olympus BX51 microscope with epifluorescence attachment and image was captured through the Applied Spectral Imaging system (Israel). The FISH result showed 94% interphase nuclei and 100% metaphase nuclei with hemizygous 22q11.2 deletion (Table 1; Figure 1). Normal control cases displayed two signals in approximately 98% interphase and 100% metaphase nuclei.

**Table 1:** is showing detailed FISH results of twins

Parameters	22q11.2 one signal	22q11.2 two signals	22q11.2 three/four signals	Remarks*
**Second Twin**				
Uncultured Blood	940	055	005	94% hemizygous deletion
Cultured Blood				
Metaphase	010	000	000	100% hemizygous deletion
Interphase	120	005	001	95% hemizygous deletion
**First Twin**				
Uncultured Blood	850	072	003	92% hemizygous deletion
Cultured Blood				
Metaphase	025	000	000	100% hemizygous deletion
Interphase	110	006	001	94% hemizygous deletion
Buccal cells	051	003	000	94.4% hemizygous deletion
Urinary Cells	025	002	000	92.6% hemizygous deletion
**Mother**				
Cultured Blood				
Metaphase	000	010	000	Normal dizygous 22q11.2 locus
Interphase	007	500	002	98.2% normal dizygous
**Father**				
Cultured Blood				
Metaphase	000	010	000	Normal dizygous 22q11.2 locus
Interphase	008	500	003	97.8% normal dizygous

*In general < 10% mosaicism with 22q11.2 interphase FISH seems clinically insignificant

### First Twin

**Figure 2:**
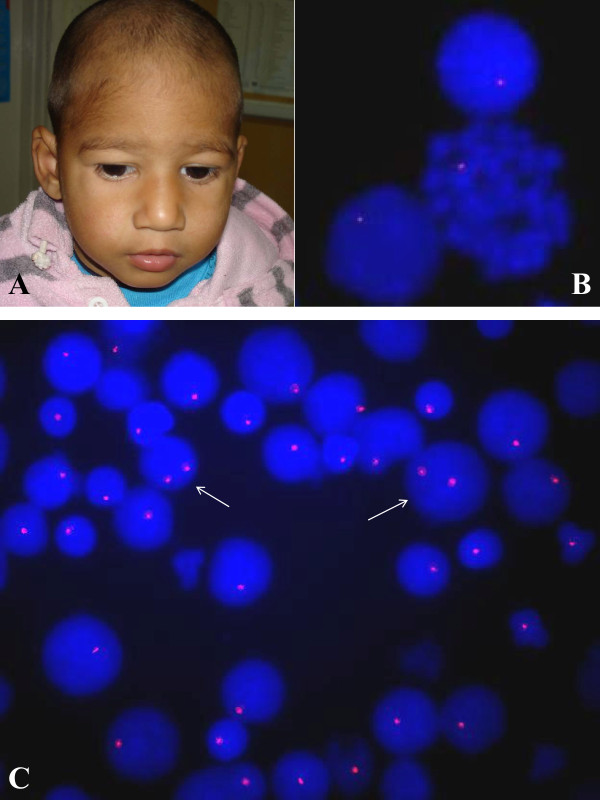
**A is showing broad nose, square shaped tip of nose, thin upper lip, wide philtrum, low set ears and mild hypertelorism and telecanthus**. B is showing 22q11.2 FISH with deletion on interphase and metaphase cells obtained from peripheral blood lymphocyte culture. C is showing 22q11.2 FISH on uncultured peripheral blood nucleated cells (interphase cells) with and without deletions (arrow).

The first twin was referred to our hospital (pediatrics outpatient department) at 13 months of age due to dysmorphic features & developmental delay and later to our department for the 22q11.2 microdeletion study. He had few episodes of upper respiratory infections, swallowing difficulties and one episode of convulsion in the first year of life. His developmental milestones were delayed, unable to speak or walk even at 13 months of age. The baby had broad nose, square shaped tip of nose, thin upper lip, wide philtrum, folded pinna, low set ears and mild hypertelorism, telecanthus & squint (Figure 2A). His weight and length was normal however, he had relatively small head (head circumference was 41 cm at 13 months i.e., below fifth percentile). His cry had nasal intonation however there was no obvious cleft palate. Ophthalmologic & auditory examination revealed no abnormality. Extensive cardiovascular work up including the echocardiography was normal. MRI scan of head and brain was also normal. There was no hypocalcemia. Conventional cytogenetics from the lymphocyte culture was normal. Since the patient had co-twin with 22q11.2 microdeletion and he has some findings suggestive of 22q11.2 microdeletion syndrome the FISH study to detect a possible deletion in the critical 22q11.2 region was done. The interphase FISH result showed 92% nuclei with hemizygous deletion for the 22q11.2 locus (Figure 2B) in the blood (mesodermal origin), 94.4% in the buccal cells (ectodermal origin) and 92.6% in the urinary cells (mainly endodermal origin). FISH on metaphase spread picked up deletion in all the 25 metaphases studied (Table 1; Figure 2C).

The twins were born to a 25 year-old mother (first pregnancy) at term by cesarean section delivery in a private nursing home. The second twin was 2400 gm at birth whereas his brother (the first twin) was 2700 gm at birth. Records of the length and head circumference at birth were not available. There was no similar problem in the family in either parental side. Parents were screened for the 22q11.2 microdeletion and were found negative for the deletion.

### Genomic DNA Microarray Analysis

DNA microarray study was carried out to find out reason for discordance using HumanCytoSNP-12 BeadChip (Illumina). The DNA was extracted from 250 uL of stored EDTA blood sample using Qiagen micro kit. The DNA quality and quantity was checked using agarose gel & Nanodrop. The concentration of DNA was normalized to 50 ng/ul. About 200 ng of DNA was used for the microarray study. The DNA sample denatured and isothermally amplified in an overnight step (whole-genome amplification uniformly increases the amount of the DNA sample by several thousand-fold without introducing large amounts of amplification bias). The amplified product was then fragmented using controlled enzymatic process using end-point fragmentation. After an isopropanol precipitation, the fragmented DNA was collected by centrifugation at 4°C and resuspended in hybridization buffer. The BeadChip was prepared for hybridization in a capillary flow-through chamber. Samples were applied to a BeadChip and divided by an IntelliHyb^® ^seal. The loaded BeadChip was incubated overnight in the Illumina Hybridization Oven. The amplified and fragmented DNA samples anneal to locus-specific 50-mers (covalently linked to one of up to 300,000 bead types) during hybridization. Unhybridized and non-specifically hybridized DNA was washed away, and the BeadChip was prepared for staining and extension. BeadChip single-base extension of the oligo on the BeadChip, using the captured DNA as template, incorporates detectable labels on the BeadChip and determines the genotype call for the sample. Image captured and analysed on iScan System. The iScan Reader uses a laser to excite the fluor of the single-base extension product on the beads of the BeadChip. Light emissions from these fluors are then recorded in high-resolution images of the BeadChip. The data from these images were analyzed using Illumina's KaryoStudio Module. The microarray analysis showed deletion in the 22q11.21 and gains in 8p23.2-23.3 & 14q11.2 in both the twins. The twins were perfect match, excepting bigger size (~0.14 MB) of deletion in the second twin at 22q11.21 locus (Table 2).

**Table 2:** is showing microarray results of twins

Positive Findings	First Twin (Milder)	Second Twin (Severe)	Remarks
*Chromosome 8*	*Gain*	*Gain*	*8p subtelomeric region*
Locus	p23.3-p23.2	p23.3-p23.2	First twin had larger size of gain (4880 bp)
Start	2190549	2190549	No differences in numbers of genes
End	2480256	2475376	
Size (~0.3 MB)	0289707	0284827	
Value	3	3	
CNV Index	0	0	
Number of Markers	33	32	
Genes	0	0	
			
***Chromosome 14***	***Gain***	***Gain***	***14q pericentromeric region***
Locus	q11.2	q11.2	Identical gain size
Start	19283777	19283777	No differences in numbers of genes
End	19494891	19494891	
Size ((~0.2 MB)	00211114	00211114	
Value	3	3	
CNV Index	1	1	
Number of Markers	14	14	
Genes	6	6	
			
***Chromosome 22***	***Loss***	***Loss***	***DiGeorge syndrome 1 or Velocardiofacial***
Locus	q11.21	q11.21	***syndrome***
Start	17257787	17118296	Second twin had large deletion
End	19792353	19792353	(139491 bp; proximal deletion)
Size (> 2.5 MB)	2534566	2674057	One gene more lost in second twin
Value	1	1	(**GGT3P**)
CNV Index	2	2	
Number of Markers	447	452	
Genes	65*	66#	

* DGCR6; PRODH; KIAA1647; DGCR9; DGCR10; DGCR2; DGCR11; DGCR14; TSSK2; GSC2; SLC25A1; CLTCL1; HIRA; MRPL40; C22orf39; C22orf39; UFD1L; CDC45L; CLDN5; LOC150185; SEPT5; GP1BB; TBX1; GNB1L; C22orf29; TXNRD2; COMT; COMT; COMT; COMT; ARVCF; C22orf25; MIR185; DGCR8; MIR1306; TRMT2A; RANBP1; ZDHHC8; LOC150197; RTN4R; MIR1286; DGCR6L; LOC375133; RIMBP3; ZNF74; SCARF2; KLHL22; MED15; POM121L4P; TMEM191A; PI4KA; SERPIND1; SNAP29; CRKL; AIFM3; AIFM3; LZTR1; THAP7; FLJ39582; MGC16703; P2RX6; P2RX6; SLC7A4; P2RX6P; LOC400891 (First Twin)

# **GGT3P**; DGCR6; PRODH; KIAA1647; DGCR9; DGCR10; DGCR2; DGCR11; DGCR14; TSSK2; GSC2; SLC25A1; CLTCL1; HIRA; MRPL40; C22orf39; C22orf39; UFD1L; CDC45L; CLDN5; LOC150185; SEPT5; GP1BB; TBX1; GNB1L; C22orf29; TXNRD2; COMT; COMT; COMT; COMT; ARVCF; C22orf25; MIR185; DGCR8; MIR1306; TRMT2A; RANBP1; ZDHHC8; LOC150197; RTN4R; MIR1286; DGCR6L; LOC375133; RIMBP3; ZNF74; SCARF2; KLHL22; MED15; POM121L4P; TMEM191A; PI4KA; SERPIND1; SNAP29; CRKL; AIFM3; AIFM3; LZTR1; THAP7; FLJ39582; MGC16703; P2RX6; P2RX6; SLC7A4; P2RX6P; LOC400891 (Second Twin)

## Discussion

We report a pair of monozygotic twins with 22q11.2 microdeletion and the discordant phenotype. The second twin presented with the classical DiGeorge Syndrome whereas the first twin presented with developmental delay and mild dysmorphic features. We have tried to find out zygosity of the twins however as one of the twin was very sick and did not provide time to investigate, we were unable to provide absolute proof for the zygosity. However, genomic DNA analysis on Illumina Beadchip 300K microarray showed that twins were perfect match except the size of 22q11.2 deletion. The microarray analysis and identical sex of the twins suggest that the twins were likely to be monozygotic. The twin research, in particular monozygotic, is a powerful tool for the study of discordances and testing hypotheses, particularly gene-environment interactions. It is generally presumed that the monozygotic twins are genetically identical and that the phenotypic differences between twins are mainly due to environmental factors. However, the genetic and the epigenetic differences between the monozygotic twins have been described [16,17]. The phenotypic variability is well known with the 22q11.2 microdeletion syndrome across unrelated, related or twins [7,18-21]. The phenotypic variability is also reported in the monozygotic twins repeatedly [6-12]. The frequent observation of variability of clinical symptoms with the 22q11.2 microdeletion in the monozygotic twins suggests underlying scientific reasons. The reasons for the phenotypic variability in the monozygotic twins could be due to various mechanisms such as the differences in size of deletion, somatic mosaicism, differences in copy number variations (CNV), differences in epigenetic changes, differences in modifying genetic factors, differences in intrauterine environment or twinning process itself.

Our likely explanation for the discordant phenotype is the differences in the size of the deletion between the twins. We have found a difference of 0.14 MB size between the twins. Although the differences in size of the deletion between monozygotic twins is unlikely however this may be possible with this syndrome as 22q11.2 locus contains several (at least eight) low copy repeat (LCR) DNA segments of more than 95% sequence homology [22]. The LCR (long stretches of repeated DNA sequence) makes the DNA unstable through non-allelic homologous recombination during cell division. The LCR take part in a homologous recombination involving unequal inter and intrachromosomal crossover, either in the meiosis or in the mitosis. This recombination leads to DNA loss or gains, thus the size of the deletion. The literatures on the differences in the size of 22q11.2 microdeletion with the phenotypic difference are contradictory, some agree [23] whereas others disagree [18,20,24-26]. Our DNA microarray analysis data showed altered size of the deletion between the twins, about 0.14 MB bigger deletion in the second twin with more severe presentation. This small difference in size of the deletion was not expected to be detectable by the FISH. Hence, we may conclude that most of the negative studies on size using FISH [18,20,24-26] may not be correct. The difference in the size with the phenotypic discordance was observed with other microdeletion syndromes viz., Yq microdeletion [27], Wolf-Hirschhorn Syndrome [28], etc. Our microarray analysis found deletion in 22q11.21 in both the twins, except the bigger size (~0.14 MB) of the deletion containing GGT3P gene (gamma-glutamyltransferase 3 pseudogene) in the second twin. The GGT3P gene initiates extracellular glutathione breakdown and catalyzes the transfer of the glutamyl moiety of glutathione to amino acids/dipeptide acceptors. The GGT3P gene increases ceruloplasmin and oxidase activity. Whether this has any role with cardiac development is yet to be linked. Thus genotypic differences i.e., the size of the deletion may be likely explanation for the phenotypic differences. The early post zygotic event could have been played a role in the alteration of 22q11.2 deletion size and, thus the phenotypic variability in the monozygotic twins.

Other possible explanation for the discordant phenotype in the monozygotic twins is the somatic mosaicism [29,30], particularly in the first twin with milder phenotype. Somatic mosaicism is usually defined by the presence of genetically distinct populations of somatic cells in an organism. Any genetic difference between the monozygotic twins represents an extreme example of the somatic mosaicism. This can result in a milder disease phenotype. The somatic mosaicism for pathogenesis may be seen as a rule rather than exception. We evaluated for mosaicism by interphase FISH by examining large number of cells and found no evidence of mosaicism in all three types of tissues (blood i.e., mesoderm, buccal i.e., ectoderm and urinary cells i.e., endoderm) in the first twin who had milder phenotype. The second twin with classic 22q11.2 microdeletion syndrome displayed approximately 92% interphase cells with 22q11.2 hemizygous deletion in blood cells. The first twin with milder phenotype displayed approximately 94% interphase cells with 22q11.2 hemizygous deletion (uncultured blood, buccal & urinary cells; Table 1). All the metaphase cells displayed 22q11.2 hemizygous deletion in both the twins. Thus, the mosaicism as underlying etiology for the twin discordance was ruled out.

The other explanation for the discordant phenotype could be the differences in the structural variations in genome. An important development in human genetics is the discovery of substantial large-scale structural variation changing the chromosomal architecture (such as deletions, duplications, insertions, inversions, and more complex rearrangements) and occurring both in phenotypically normal as well as abnormal subjects. The most explored subtype of structural variation involves changes affecting copy number of DNA segments (denoted as copy number variation or CNV), often involving fragments of chromosomes that are considerable in size. In recent years genomic studies using microarray platform provides genomic explanation (CNV) for phenotypic discordance in the monozygotic twins in schizophrenia [31,32], parkinsonism/dementia [10], etc. It is now well known that the monozygotic twins have CNV leading to differences in the phenotype [10,33]. The CNV have shown to confer increased risk for the congenital cardiovascular disorders such as the tetralogy of Fallot [33]. Similarly, in a mice experiment with low copy transgenic manifest ear disorders only, while the mice with high copy transgenic presents with reduced viability and multiple anomalies that were similar to humans VCFS/DGS [34]. Our microarray analysis did not find any discordance related to the CNV between the twins.

The discordant phenotype could also be due to the differences in the epigenome [**7**]. The epigenomic changes (e.g., DNA methylation) are able to influence the expression of the gene without affecting the DNA sequence. The methylation of genomic DNA may affect a variety of processes related to gene expression including imprinting, chromosome inactivation and gene silencing. Given the variety of developmental anomalies associated with 22q11 deletions, it is logical to implicate a methylation difference between the twins that would alter the expression of some/most genes of this region. We are unable to comment on this hypothesis at this time due to various technical difficulties.

The discordant phenotype could also be due to the early post zygotic second hit (mutation) in modifying genetic factors (e.g., fgf8 at 10q24, fgf10 at 5p12-p13, Gbx2 at 2q37, Pitx2 at 4q25, Vgef at 6p21.3, Tgfβ at 1p34.1; Chordin at 3q27, Sonic hedgehog at 7q36, etc) in one of the twins [9,35-40]. The second hit (mutation) hypothesis may entail a variety of mutational mechanisms including replication errors, base changes and additional deletions involving LCR and Alu repeats of the region. The second somatic hit hypothesis, however, need not be restricted to the genetic changes at the level of the DNA sequence but it may also involve the epigenetic changes. Stalmans et al [36] provided evidence that variation in the gene encoding vascular endothelial growth factor may modify the cardiovascular phenotype with hemizygous for the 22q11.2 deletion. Similarly, Driscoll et al [37] reported modifiers for palatal phenotypes with this syndrome. It is known that the genetic background influences the penetrance of cardiovascular, thymic, and parathyroid anomalies in the mice [38,39]. Girirajan et al [41] proposed a 'two-hit' model, wherein a secondary insult is necessary during development to result in a more severe clinical manifestation. The second hit could potentially be another CNV, a disruptive single-base-pair mutation in a phenotypically related gene or an environmental event that influences the phenotype or altered deletion size as with our twins. The two-hit model also helps to explain the underlying phenotypic variability reported for several recurrent microdeletions. The majority of second hits are probably not detectable even by very high-resolution arrays. Whole-genome re-sequencing may reveal a surprising number of additional contributing loci.

Finally, the intrauterine environment or the twinning process [11] itself, and non-genetic factors [42] may influence phenotypic discordance. The twinning process imposes a growth disadvantage that may be more severe in one [8] thus may account for the discordance of malformations in the twin pairs. Other influences of the twinning process, which cause discordant cardiovascular anomalies include the disturbance of laterality and the placental vascular anastomoses.

We conclude that altered size of the deletion may likely be the underlying etiology for the discordance in the phenotype in our monozygotic twins. We think early post zygotic mitotic non-allelic homologous recombination could have been played a role in the alteration of the size of 22q11.2 deletion and, thus the phenotypic variability.

## Consent

Written informed consent was obtained from the parents of patients for publication of cases and accompanying images. A copy of the written consent is available for review by the Editor-in-Chief of this journal (additional file 1).

## Competing interests

The authors declare that they have no competing interests.

## Authors' contributions

AH formulated activity plan, checked & interpreted results, besides being the Principal investigator of the project funded by Indian Council of Medical Research, India. He also reviewed clinical findings, prepared manuscript and responded to the queries of reviewers. He is the guarantor of the manuscript. MJ carried out all FISH related activity under guidance of AH, besides working as Research fellow for the project under AH. IC also carried out FISH related activity under guidance of AH. BV was involved in microarray analysis & interpretation of microarray data. All authors read and approved final manuscript.

## Supplementary Material

Additional file 1 **Consent of parents**. Consent for permission of using photograph, clinical & other information for publication. Click here for file
