# Supplementary material for: Chromosome 22q11.2 microdeletion in monozygotic twins with discordant phenotype and deletion size
Source: Mol Cytogenet. 2012 Mar 13;5:13. doi: 10.1186/1755-8166-5-13 (PMC3325853; doi:10.1186/1755-8166-5-13)
Supplement: Additional file 1 — Consent of parents. Consent for permission of using photograph, clinical & other information for publication. [file 1755-8166-5-13-S1.PDF]

Consent Form

For patient's consent to publication of material in
Any Journal/Book/etc

Clinical Information or photograph or Test results or All or Any

Date: 09-03-2010

I/we give my consent for above materials to be used for publication/presentation/etc

Please tick as appropriate:

I am the patient
I am the patient's relative/parents
I understand the following:

(1) The material will be published without my/the patient's name attached and every attempt will be made to ensure my/the patient's anonymity. I understand, however, that complete anonymity cannot be guaranteed. It is possible that somebody somewhere - perhaps, for example, somebody who looked after me/the patient if I/the patient was in hospital or a relative - may identify me/the patient.
(2) The material may be published in Journal/Book/any other mode
(3) The material will not be used for advertising or packaging.

Signed: [signature] Pooja Verma

Name in BLOCK CAPITALS: HARISH KUMAR (Father)
Pooja (mother)
